# Storytelling in Scientific Conferences: Mitigating Misinformation Risk

**DOI:** 10.3389/ijph.2024.1607289

**Published:** 2024-04-16

**Authors:** William B. Weeks

**Affiliations:** AI for Good Lab, Microsoft, Redmond, WA, United States

**Keywords:** misinformation, misinformation related to health, statistical analysis, COVID-19 vaccine, adverse drug reactions

## Storytelling

Politicians frequently use anecdotes to drive home a point. They describe an encounter with an individual that characterizes an issue that their opponent opposes, for which they have a policy response, and that plays to their audience’s emotions. The factual nature of the encounter is unclear: in retellings, details may differ.

I have noticed a trend for presenters in scientific conferences to mimic this approach. Introducing their scientific work with a story that is purportedly a case description, they articulate what is presumed to be a common plight, one that motivates their research, initiative, or policy proposal. While such stories may be more common in public health presentations, where social determinants of health are reckoned as drivers of the health concern the patient faces, stories are seemingly increasingly used in a variety of scientific and policymaking conferences to articulate the commonality of the constellation experiences that they represent.

These stories are entertaining, engaging, and relatable. But I fear that they can serve three potentially nefarious purposes.

First, the stories imply causality. While statistically supported causal relationships between the story’s protagonist and the presumptive circumstances associated with the outcome of interest are not clearly articulated, the story implies that they exist and are strong. Humans are storytellers, and good story suspends disbelief; however, with objectivity as their distinguishing characteristic, scientists need to maintain a healthy skepticism about what is presented to them and should demand statistical validation that supports proposed relationships.

Second, the stories normalize what might not be normal. While good anecdotes describe a policy gap, the protagonist may not be representative of the study’s demographic or clinical characteristics. Indeed, articulated specific circumstances might be unique and better serve as the subject of a morbidity and mortality conference wherein particular, often rare, circumstances align to generate unanticipated outcomes. What is presented as commonplace might be extraordinary.

Third, the stories can serve as fodder for misinformation or disinformation campaigns. Without statistical support and with presumed representativeness, stories, particularly if amplified on social media, might sway public opinion. Social media is ubiquitous, recorded conference material is easily accessible, and, in an age wherein laypersons want to do their own “research,” influencers—and those seeking to sew discontent—might select a story to support extreme views, perhaps those not intended by the storyteller.

## A Set of Stories, as Examples

A set of stories illustrates different approaches.

When commenting on the 2021 Met Gala’s COVID vaccination requirement, Nicki Minaj, a Trinidadian rapper, tweeted an anecdote about her cousin’s Trinidadian friend whose COVID vaccination presumably caused his testicles to swell, sudden impotence, and cancellation of imminent wedding plans by his fiancée [1]. The story was entertaining, engaging, and relatable: it may have convinced readers that COVID vaccines were dangerous and had previously unreported side effects that might adversely impact body morphology, sexual functioning, and, presumably, marriage.

In contrast, a recent scientific report revealed that a 62-year-old German man who had received 217 COVID vaccinations in a 29-month period (130 of which were obtained over 9 months) experienced no vaccination-related side effects and did not alter “the intrinsic quality of adaptive immune responses.” [2] This objective study used statistical methods to support hypotheses and causation to explore the medical sequelae of the very rare (and even illegal) circumstance of monumental overuse of a medical service.

Both stories had a brief viral presence on social media, neither represents normative behavior or findings, and only one includes objective evaluation of confirmed factual material.

To be sure, Nicki Manaj is a singer, not a scientist: one would not expect her to use a scientific approach to evaluating potential COVID vaccine side effects. Regardless of any factual support of the cousin’s friend’s experience, her story exemplifies a layperson’s potential confusion between association and causation, the potential normalization of an exceedingly rare event, and the possible use of a story for nefarious purposes.

Perhaps equally extraordinary, the story of the over vaccinated German avoided hyperbole, stated facts, and used the scientific method to derive conclusions.

## A Proposed Solution

**FIGURE 1:**
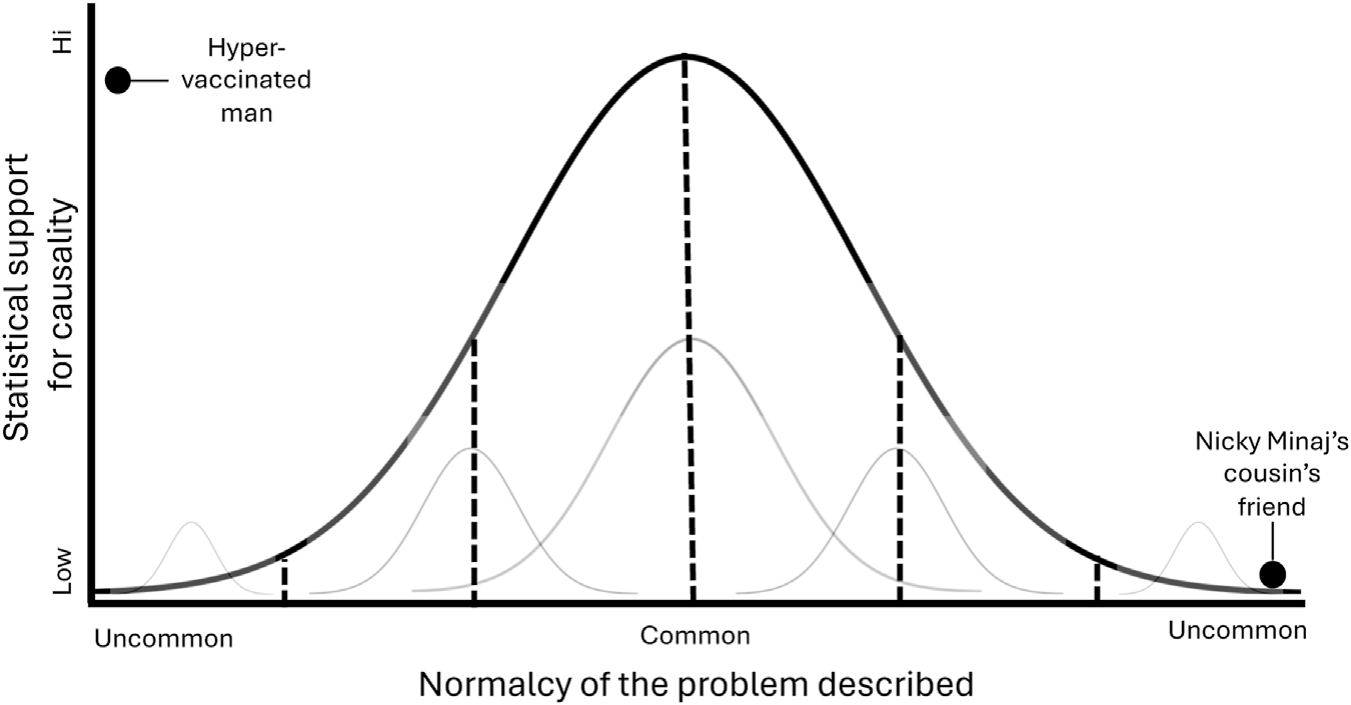
An example of how stories might be contextualized across two dimensions: statistical support for causality of the presented relationship and the normalcy of the problem described (whether the story involves adverse reactions to vaccination or to social determinants of health) (United States, 2024).

To avoid amplifying sensationalism, implying causation where there is none, and the inadvertent foment of misinformation campaigns, I propose using a pre-emptive solution to storytelling in scientific conferences: when a story is presented, situate the protagonist’s story within a series of normal curves and on two axes: statistical support for causality of the presented relationship and normalcy of the problem described (Figure 1).

Much of human experience and behavior falls within a bell curve; even at the extremes of the bell curve, there are distributions. Indeed, scientists often study those extremes—whether as case studies, very rare diseases, or adverse events to vaccinations. While the scientists may attempt to generalize findings to broader populations, their manuscripts should include limitations that study findings apply to the cohort studied and may be different in other populations. In medicine, science, and life, rare events are rare: social media hungry audiences should understand the representativeness and rarity of the information that they consume.

Storytellers in scientific conferences should adhere to the scientific method, fairly and objectively present data, and describe the population they studied. Just as with a required conflict of interest statement at the beginning of a presentation, this proposal provides an easily visualized way for a scientist to convey the representativeness of the examples used in their stories.

Doubtless, even with such contextualization, social media influencers and misinformationalists will select and amplify content that supports their goals. But in scientific conferences, when scientists use stories as useful motivators and engagement tools, they should articulate the degree to which they are generalizable to the study population and is not a description of a black swan event.

### Conclusion

Stories can be motivating, engaging, and relatable; they can provide context that highlights the scientific point to be made; and they can make key points memorable. But, in science, stories should be representative of the population being studied. Our world is increasingly torn by extremism that is amplified by sensationalism; scientists should adhere to the objectivity that defines their discipline.
